# ConTemplate: exploiting the protein databank to propose ensemble of conformations of a query protein of known structure

**DOI:** 10.1186/1471-2105-15-S3-A5

**Published:** 2014-02-11

**Authors:** Aya Narunsky, Nir Ben-Tal

**Affiliations:** 1 Department of Biochemistry and Molecular Biology, George S. Wise Faculty of Life Sciences, Tel Aviv University, Tel Aviv, Israel

## Background

**Figure 1:**
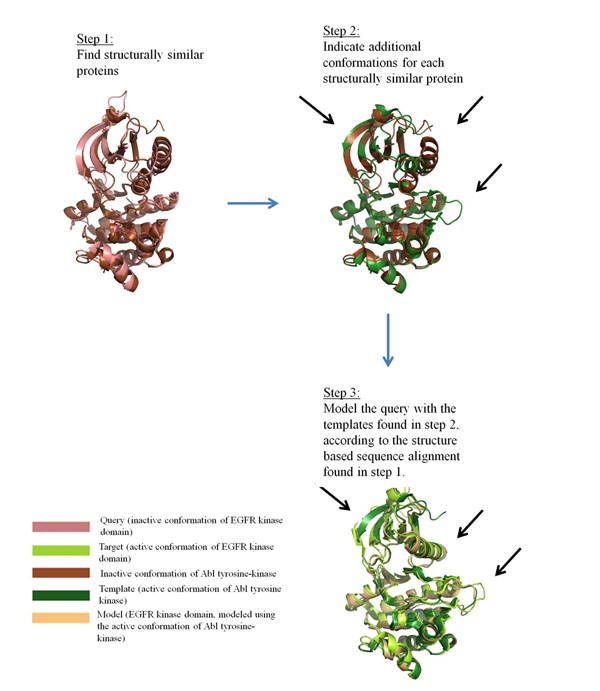
ConTemplate methodology, demonstrated using the known structure of the EGFR kinase domain in its inactive conformation as a query and reproducing its active conformation; the RMSD between the active and inactive conformations is 4.17Å. **Step 1:** Selecting proteins with structural similarity to the query; only one is shown here. **Step 2:** Finding alternative conformations of the proteins detected in Step 1. The black arrows mark the regions with the main differences between the conformations. **Step 3:** Modeling putative new conformations of the query using the conformations detected in step 2 as templates; only one is shown here. The black arrows indicate the similarities between the model, template and actual known conformation in the main regions of the conformational changes.

Proteins often alternate between several conformations, e.g., active and inactive states of receptors, open and closed states of channels, etc. However, in many cases only one conformation is known. The prediction of additional (biologically-relevant) conformations of a protein can provide more insight into its function in health and disease. We introduce the ConTemplate computational tool for modeling putative conformations of a query protein with (at least) one known conformation by assuming that pairs of structurally similar proteins may also share similar conformational changes. A three-step procedure is used (Fig. 1): First, the protein databank [1] is searched for structurally similar proteins to the query [2]. Structure-based pairwise sequence-alignments are built between the query protein and each of the structurally similar proteins. Second, other known conformations (i.e., different from those resembling the query) of these proteins are indicated [3]. Third, by using the alignments found in the first step, and modeling on the structural templates found in the second, ConTemplate suggests new conformations for the query protein.

## Results

We demonstrate the method with the kinase domain of the Epidermal Growth Factor Receptor (EGFR). Using the inactive conformation as our query, we reproduce the active conformation [4] with root mean square deviation (RMSD) of 1.76Å, based on the query's structural similarity to the inactive conformation of Abl tyrosine-kinase [5], together with the known active conformation of the latter kinase [6]. The sequence identity between the two kinase domains is only 40%, and the fact that they share similar active and inactive conformations might not be obvious.

## Conclusions

The idea of inferring new conformations of a protein of interest based on known conformations in related proteins is not new. However, to the best of our knowledge, ConTemplate is the first automated implementation of this approach.
